# Manufacturing Options for Activated Carbons with Selected Synthetic Polymers as Binders

**DOI:** 10.3390/ma17081753

**Published:** 2024-04-11

**Authors:** Iwona Skoczko, Ewa Szatyłowicz, Adam Tabor, Remigiusz Gumiński

**Affiliations:** 1 Department of Technology in Environmental Engineering, Faculty of Civil Engineering and Environmental Sciences, Białystok University of Technology, St. Wiejska 45A, 15-351 Bialystok, Poland; i.skoczko@pb.edu.pl; 2 Grand Activated sp. z o.o., St. Białostocka 1, 17-200 Hajnówka, Poland; tabora@grand-activated.pl; 3 GreenTurf Sp z o.o., St. Krzysztofa Kolumba 88-89, 70-035 Szczecin, Poland; remigiusz@guminski.pl

**Keywords:** adsorption, activated carbon, AC binders, carbonization, activation

## Abstract

Formed activated carbon (AC) is a multipurpose product with developed adsorption properties that is widely used in various areas of life. To create AC, hard coal has to go through various processes: grinding, granulation, carbonization, physical and/or chemical activation. Presented research was conducted in the professional company manufacturing activated carbons. Studied AC reached the demanded shape of grains thanks to binders added to granulation process. Research on the AC formed using new polymeric binders (applied so far in other branches: pharmacy and construction materials) is presented in this manuscript. Tested binders were not used before to manufacture ACs in the professional technological line. Such polymers as: sodium carboxymethylhydrocellulose (CMHC), poly[1-(2-oxo-1-pyrrolidinyl)ethylene] (POPE) and enriched methyl-hydroxypropyl cellulose MHPC were studied in this work. Conducted research has proven efficiency of 8% CMHC which allowed for proper granulation and carbonization and reached the best parameters. Single- and double-stage activation was investigated for AC with this binder. For newly manufactured AC BET surface and pore volume increased accordingly from 774 m^2^/g and 0.58 cm^3^/g (1-stage) to 968 m^2^/g and 0.72 cm^3^/g (2-stage). Chemical elemental features of surface of the best AC showed beside elementary carbon also calcium, silicon and aluminum ions as well as groups with an acidic character, phosphates, sulphates and chlorides. The new AC had a higher Mechanical Strength reaching 99.9% and a lower Ash content and Volatile Matter than AC manufactured with previous binder—molasse. The new AC is intended to be directed for full production line and implementation to usage after positive certification. It may be useful in water treatment. It will also find application in the treatment of industrial and municipal wastewater.

## 1. Introduction

Activated carbon (AC) is a multipurpose product widely used in various areas of life. Its adsorption properties ensure harmful substances removal from all types of media in liquid or gaseous form. The porous surface makes the ratio of activated carbon’s filtration area to its mass enormous and can reach up to 1000–4000 m^2^/g [1,2,3,4]. Purification process based of adsorption lets to bind impurities to AC’s outer surface. As a result, the contaminant (from the fluid or gas) are trapped in active centers on the external or internal surface of the AC [5]. Activated carbon purifies a variety of media without the use of additional chemicals and is therefore it is safe for human and environment [5,6]. It can be used at home, office, car maintenance, gas purification, masks and gas filters. It also allows the effective purification of aqueous solutions from toxic, taste- or odor-generating substances and other undesirable compounds, finding application in, among others, the food industry (e.g., sugar factories, distilleries, breweries, soft drink production, confectionery production, dietary fats, etc.) or the pharmaceutical industry (patches for hard-to-heal wounds). Its adsorptive properties help remove harmful substances from the digestive system too [6]. This product can also find use in cosmetics. It is commonly used for the purification of drinking water and the wastewater treatment. Thanks to its structure, it is able to absorb undesirable substances from water, such as heavy metals, phenols, dyes, drugs and toxins. AC is characterized by: effective mechanical filtration, very high porosity and much higher absorption efficiency than other filtration materials [7,8,9,10].

The literature [11,12,13,14] states that activated carbon can be manufactured from a wide variety of carbon-containing raw materials, such as coal, lignite, wood, peat or coconut shells. The process of converting the raw material into the final product consists of a thermal process and a chemical process as: drying, grinding, granulation, carbonization (pyrolysis), physical and/or chemical activation, final washing and drying. One of the most important manufacturing processes is granule activation in temperatures of 700–1100 °C in the atmosphere of water steam or carbon dioxide. The water-gas reaction, plays a significant role, as a highly porous, active coal skeleton is formed by partial gasification (1).

C + H_2_O → CO + H_2_ (1)

The processed activated carbon is distributed in loose, granulated, molded or dusty form [12]. Granular activated carbon (GAC) is the most commonly used and desirable form of carbon. The porous GAC particles interact with the entire surface of the flowing water, while the retained contaminants are easily removed, allowing the product to be used for many years [15]. Granules are created to increase the homogeneity of the AC, its density (less volume per unit weight), for easier storage and transport, to simplify dosing, reduce ash content and to improve general appearance of the product [16]. Wet granulation uses a granulation liquid (binder & solvent) to support agglomeration by forming a wet pulp by adhesion. Wet granulation is the most common formulation technique, even though it involves many unit processes such as wet forming, drying and screening, which are complex, time-consuming and also costly. Granules are created by mixing AC with liquid and binder [17,18,19] in a closed system with low moisture. It allows the preparation of highly compressible materials and also the modification of low additive properties. In addition, the technique provides granules with larger particle size, good flow properties and high tensile strength, which can be directly compressed into pellets with sufficient hardness and low friability. The limitations of this technique are the requirements for significantly high energy inputs. Binders are a key formulation ingredient in wet granulation, providing adequate surface wetting ability to ensure adhesion and cohesion between inter-particle surfaces in the wet state, as well as excellent plasticity, density and bonding ability [17]. As binders there may be used products from the thermochemical processing of coal, lignite and crude oil, such as post-production tars, pitch and asphalts. Nevertheless the most commonly used binders are organic, plant-derived substances such as lignans, cellulose, starch or resins. Synthetic substances such as polymers are also used if they have suitable viscous properties during the production process. By-products from the wood and food industry, such as prepared hardwood tars, post-sulphite lye, sugar molasse, corn syrup or starch, also play a significant role in this field [20,21]. The choice of a particular binder depends on many factors, such as the type of raw material, technological specifications, as well as the desired properties of the final activated carbon. The properties of polymeric binders with relation to compressibility, density, matrix-binder interaction, etc. in solid dosage forms have been extensively studied for wet granulation too [19,22,23,24,25]. Different binders can affect porous structure, adsorption capacity and other final AC characteristics. Even after careful selection of binders, granules not always have optimum pelleting characteristics such as powder fluidity, granule strength, compact ability, friability, disintegration time etc. [18].

Under laboratory conditions, the granulation process can be carried out gradually and with low efficiency, which usually leads to shaped granules with most binders. The quality of the final product is determined by the sorption and strength parameters of the raw coal. Experimental works on a single coal-pulp granulation has been the subject of numerous research papers. The literature does not show publications describing the professional production of activated carbon on a real scale including all necessary manufacturing processes.

Therefore, within this work, a number of tests were carried out, initially under laboratory conditions and then in a real manufacturing scale of the technological AC’s production line: from the processing of the raw material, through all the production stages, up to the formation of granulated activated carbon. The main goal of the study was to create the new granular activated carbon with optimal porosity and mechanical strength with the best binder (from the group of various synthetic polymers) in the wet granulation process. In this manuscript there are presented research results on the new product which after positive certification may find its way to the real trade market. This product is activated carbon formed with new binder. The most popular binder in the past was molasse– but today molasse become difficult to reach for AC processing purposes. What is more molasse is unstable in production’s conditions and requires to often change technological parameters. Conducting presented in this manuscript experiments, new binders used in pharmacy and construction materials formation were tested. These binders were not used so far to manufacture ACs in the real technological line.

## 2. Materials and Methods

The research was carried out at the Grand-Activated Ltd. manufacturing plant—producing activated carbons and located in Central Europe. The company specializes and focuses on the manufacture of different activated carbons with wide range of applications. Experiments were supported by Bialystok University of Technology to moderate technological line production and improve the activated carbon parameteres using new binders.

### 2.1. Materials

Laboratory tests were carried out and batch production trials of granular activated carbon was conducted using cellulose polymer-based (Table 1) binders:

1. Sodium carboxymethylhydrocellulose (CMHC) (Ashland, OR, USA) is refined, water-soluble, high viscosity (1500–2500 mPa*s) anionic polymer that acts as a thickening agent, rheological modifier, binding agent, stabilizer, protective colloid, film-forming agent and water retaining agent. It is patented (Patent No. 4,919,711) as bonding agent for metal ore in metallurgy. CMHC is widely used in toothpastes as a water-binding thickener to prevent syneresis and to impart a semi-fluid state to various paste and ointment formulations. CMHC is also used in creams and adhesive powders for dental prostheses. The preparatory steps for this work assumed the use of CMHC with a high fineness and its uniform wet mixing with carbon [26].
2. Poly[1-(2-oxo-1-pyrrolidinyl)ethylene] (POPE) (Ashland, OR, USA) is a water-soluble polymer made from N-vinylpyrrolidone monomer by free radical polymerization. POPE’s varied solubility in aqueous and organic solvent systems, combined with its non-toxic nature, are key properties that provide POPE with excellent flexibility. It is mainly used in the pharmaceutical and cosmetic industries as a filler, increasing the volume of the cosmetic product. As a binder, it ensures the binding of cosmetic product ingredients and tablet formation. Its non-toxic and supports corneal and conjunctival treatment in ophthalmology. Not previously used in the production of activated carbons [27].
3. Methyl-hydroxypropyl cellulose (enriched)—MHPC (Ashland, OR, USA) has higher molecular weight than construction binders containing crystalline cellulose. Used as a versatile binder in protective coatings and for clays granulation and extrusion of ceramics. Its use in the molding of bulk products allows it to maintain the shape of molds, e.g., extruded ceramic products. It is also an effective water retaining agent [16,28].

A medium-grained hard coal with a diameter of approx. 8–31 mm was selected to carry out research into the production of formed activated carbon with polymer binders. It is a coal with a high volatile content (approx. 28%), high caking ability (approx. 7%) and low ash content (approx. 5%).

#### Methods of Solution Preparation

CMHC: Wet granulation was used. The CMHC binder was introduced in a dry crystal form. Granules involving a granulation step were less sensitive to the use of possible and/or additional binder excipients than in direct compression. In our tests, we used the method of direct compression. The original particle structure remains largely unchanged, so individual particle and excipient properties have a more direct impact on the formulation performance, such as consistency and compactness, and determine the success of granule formation.

POPE: The use of POPE solution as a binder helped to eliminate the influence of factors such as moisture and heat for special AC purposes (i.e., AC for medicine). Water was beneficial in increasing the dissolution of POPE. There was used POPE in wet granulation. Its application in dry granulation let to directly press processing AC to tablets for gastric problem. This method unfortunately blocked hydrophilic features of final product. In experiments described in this paper there was used wet granulation for AC better formation effect and hydrophilic features (compulsory for water/wastewater treatment).

MHPC: This product is soluble in water (10 mg/mL). However, it is very important to thoroughly disperse the particles in water with mixing before they dissolve. Otherwise, they will lump and form a gelatinous membrane around the internal particles, blocking water absorption. We added dry mixture to water with agitation and mixed until the product was completed hydrated and the solution—consistently smooth.

### 2.2. Manufacturing Process

Tests on the formation of activated carbon using the investigated polymers were conducted under laboratory conditions and on the real production line in our company. The activated carbon production process included unit processes such as:

1. Grinding of hard coal and pulverization in industrial mills. The same grinding equipment was used in laboratory and industrial tests.
2. Molding of the binder-and-coal-dust paste (prepared in the previous stage); the process was carried out in industrial mixers. The same grinding equipment was used in laboratory and industrial tests.
3. Granulation, granules were formed from coal-binder paste; process was carried out in industrial granulators using extrusion elements allowing to obtain granules with a diameter of 4.2 mm. The same grinding equipment was used in laboratory and industrial tests.
4. Having dried the granules and pre-hardened their surface, the shaped granules were sent directly to a rotary drum dryer heated by the gas from the carbonization furnaces. Drying was performed at temperatures of 200–400 °C. The same drying equipment was used in laboratory and industrial tests.
5. Carbonization of the dried granules was carried out in a laboratory carbonization furnace (lab tests) and in industrial rotary carbonization furnace (technological tests). Carbonization was carried out at a temperature of 800 °C for a period of 60 min. The rotational speed of the furnace was programmed to take 1 rotation for 30–35 s.
6. Activation was carried out in laboratory and industrial activation furnaces. Primary activation process was performed only in laboratory conditions in temperature 900 °C and time 120 min. The activating agent was carbon dioxide produced in the activation furnace which was recycled from emitted fumes. An activation in industrial condition was tested as double-stage activation process. The first stage was completed at 900 °C and the second at and 600 °C. The activation time for both stages was 120 min. The activating agents in the process were water steam (H_2_O) and carbon dioxide (CO_2_). CO_2_ was supplied to the furnace as a waste product from the carbonization furnace.

Preparation of the paste from the binders and coal dust was carried out in industrial mixers. Every binder-coal batch was prepared from 3 kg of coal. Then tested synthetic binders were mixed with different volumes of water to check the best ratio: 800 mL, 900 mL and 1200 mL and added to coal. There were tested different doses of binders:

- POPE and MHPC were added to mixer in a dose: 30, 150, 240 g/3 kg of coal,
- CMHC was tested in doses: 30, 150, 240, 360, 450 g/3 kg of coal.

In this work, 11 laboratory and industrial granulations were carried out using the cellulose polymers described in Section 2.1, taking into account the different concentrations of tested binders (Table 2).

### 2.3. Analytical Methods

In the AC production process the parameters of the activated carbon were controlled, as shown in Table 3. Wider description of analytical methods is included in “Supplementary Materials”. Laboratory testing of the carbon parameters was carried out in our company’s analytical laboratory. The determinations of properties and parameters at Grand Activated Ltd. were specifically designed for scientific research.

#### Sample Imaging Using a Scanning Microscope

Microscopic observations of the filler were performed using a Scios2 DualBeam FIB-SEM microscope, Termofisher, Scientific (Walham, MA, USA). An accelerating voltage of 2 kV was applied during observations. Magnifications ranging from 500 to 5000× were used during observations.

## 3. Results and Discussion

The purpose of conducted research described in this manuscript was manufacture the new AC which may replace popular WG-12. WG-12 was manufactured by the Grand Activated Ltd. company and commonly used for water and wastewater treatment. Due to its high Specific Surface Area and developed pore structure, activated carbon WG-12 let to remove from water: organic pollutants, pesticides, detergents and a number of micropollutants harmful to health. It is also used to dechlorinate water and improve its taste and smell. Its manufacturing based on molasse as binder which become today difficult to reach because of inflation and difficult supplies from domestic producers. What is more molasse is usually unstable in production’s conditions and require to change often technological parameters. For this reason, efforts have been made to manufacture AC having similar features as WG-12. Tests have started on the experimental production of AC from coal and new binders in place of molasse. The newly manufactured AC is planed (after positive certification) to direct to water treatment, both in large water supply stations, in small filter and container installations. Therefore tests were carried out on the use of other polymeric binders (Table 1). These binders have not been used in AC’s mass production so far and belong to the group of compounds that bridge construction materials or support tablet formulation in the pharmaceutical industry.

### 3.1. Granulation Process

In order to produce each of the coal-binder paste presented in Table 2, the same technological production conditions were applied (processes mentioned in Section 2.2). The pulverised raw hard coal was mixed with water and binder in a special industrial mixer by introducing dry ground coal, dry binder and water, respectively, to liquefy the mixture until it reach the consistency of a homogeneous paste.

**Figure 1:**
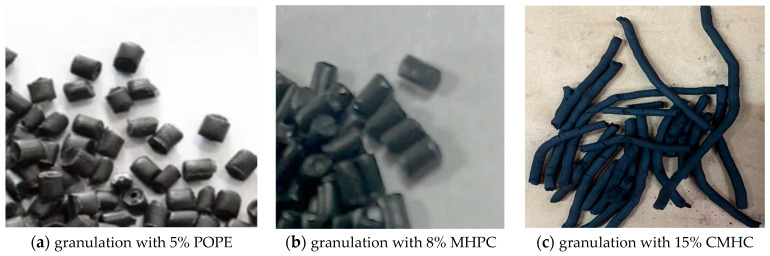
Appearance of granules leaving the granulator with different binders: POPE, MHPC and CMHC.

According to Neimark’s group [29] binder’s viscosity is one of the most important parameters and also decisively influences the granulation effect and subsequent granule strength. The team of Mills [30], on the other hand, found that a minimum viscosity of 10 mPa was necessary for successful granule formation. Further increase in viscosity has a beneficial effect on granulation up to a certain critical value, exceeding which the opposite effect is observed. Such a situation was observed in described in this paper research, where binders with high viscosity POPE and MHPC did not allow efficient formation of carbon granules, and the critical value turned out to be a POPE viscosity of 5000 mPa. The lowest viscosity had CMHC, 2000 mPa, and this proved to be an effective for bonding carbon granules. In his work Mills et al. [30] wrote that granulation of loose materials using low viscosity binders is possible due to the dominance of the layered growth mechanism. Granulation carried out with higher viscosity additives relies on a process of coalescence and lack of layered material to maintain granule sphericity. A similar relationship was observed in own research, where the forms produced in the granulation process were unshaped and affected by crushing and disintegration. The composition of the paste is given in Table 2. The pastes of the pulp of coal and the all tested binders had the proper consistency, clumping when lightly squeezed in the hand, which is typical for raw granules. After leaving the mixer, the paste was transported to the granulator. It was observed that the formed granules using polymers concentrations of 1% and 5% were unshaped, short (Figure 1a), fragile, became quickly deformed, and also easily self-agglomerated into larger conglomerates (Figure 1b). Only higher concentrations of used polymers allowed the required granules to be formed. With 15% CMHC, the granules were long (Figure 1c), but did not break when dropping out of the granulator.

The positive effect of hydroxypropyl cellulose may be confirmed by Rajniak and team [31]. They investigated the granulation potential of raw material with HPC concentrations of 5, 10, 15, 20, 25, 30% and found that the physical properties of aqueous solutions of HPC binders with different HPC concentrations had a direct effect on the formation and properties of the granules.

### 3.2. Carbonization Process

During carbonization (a.k.a. charring or pyrolysis) process, AC pellets undergo thermal degradation in an inert gas atmosphere. In conducted experiments the process temperature used was approximately 800 °C, although Plavnies et al. [32] and Sultana et al. [33] report that typically AC pyrolysis is carried out at lower temperatures, i.e., around 400–600 °C. Coal-based raw materials must undergo a carbonization process to remove moisture, and to get rid of any volatile substances that are products of the transformation of coal at elevated temperatures. Then the AC may be subjected to activation processes.

There was noted that coal carbonization, which is the first step of its thermal processing, is the basis for achieving the required adsorption and strength parameters of the final product. It is well known [34,35,36,37,38] that hard coals are good raw materials for the production of activated carbons, as they allow to produce a highly porous internal structure during carbonization and activation processes. At the same time, it requires precise adjustment of the process parameters (time, temperature, composition and quantity of the processed charge). Plavniece et al. [32] noticed that for the granular carbons hardening, pyrolysis is more difficult to implement than for pulverized or grainy carbons (primary unprocessed). The pyrolysis of shaped granules requires careful processing due to the need to depolymerize organic components and synthetic additives into the original carbon structure. As an example of proper substrate preparation for the production of formed activated carbons, Ruiz et al. [38] used mixed bituminous and anthracite coals with a high content of vitrinite (the basic hard carbon material) rich in lignin and cellulose (with low volatile matter and ash content). After carbonization, the mixture showed a low degree of anisotropy and a lack of plasticity. The researchers concluded that bituminous coal has a lower degree of carbonization than anthracite and a high volatile content, that is why it can be more easily subjected to pyrolysis and gasification.

**Figure 2:**
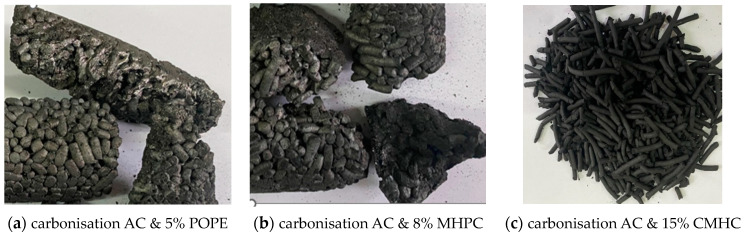
General appearance of formed granules of AC after carbonisation.

In conducted experiments there was used formed activated carbon from coal pulp and a polymer binder. No depolymerization occurred in the carbonization furnace and the pellets liquefied during the process. The charcoal was conglomerated for most samples and not suitable for further use in the thermal activation process (Figure 2). The literature [29,30,31] reports that anisotropic coals may be formed in the presence of tars and carbons. These materials aggregate during carbonization and may form a semi-liquid form. The transformations result in the formation of liquid crystals during the coals pyrolysis. From this reason the temperature of the process is of great importance. Table 4 shows the properties of the charcoals after the carbonization process. Only samples # 6, 8 and 9 including bonding additives in the form of CMHC (15%), CMHC (8%) and CMHC (12%), could be targeted for further experiments. The other tested synthetic polymers, i.e., POPE and MHPC, failed to produce charcoal in the form of free-flowing granules. Further processing was not possible. Among the non-agglomerated samples, the sample prepared with 8% CMHC had the lowest ash content, while the sample prepared with 12% CMHC had the lowest volatile matter content. All non-agglomerated samples had a high mechanical strength of 99.9% and could be subjected to the activation process. As mentioned before, in the carbonization process, volatile parts are released from the raw carbon material and the structure of activated carbons is shaped. Literature [7,13,32,33,34] additionally informs that in carbonization process develops an internal pore network, which cannot be blocked by the semi-liquid bonding material. Carbonization has to prepare processed ACs to activation stage where activating gas should have easy access to the entire volume of the carbon granule.

### 3.3. Activation Process

Studied in this work activated carbon pre-processed in the carbonization was modified by reaction with the supporting substance in the activation process. The final result was a product with a developed internal structure and high porosity, which after thermal processes is washed, dried and can be used as activated carbon. Activation of carbon can be physical and/or chemical [10,23,39]. In physical activation, to achieve the expected porosity, the most common reactants are: water steam (which is the most common and widely used agent), sometimes also air, CO_2_ and oxygen. In full technological line, due to high investment costs, oxygen is rarely used on an industrial scale [33]. However, prior to physical activation, some carbonaceous materials require additional preliminary processes to achieve the appropriate porosity and pore structure, as well as to develop the final shape of the forms and size of the processed particles, grains or granules. Pogorzelska [12] states that due to the wide range of feedstock used in activated carbon production as well as the variety of reactants and activating substances, different types and forms of activated carbons can be obtained.

Conducted experiments on activation started in the industrial laboratory (Table 5). In the ACs activation one of the most important factors is process’s temperature. There was carried out experimental laboratory activation process at 900 °C for 120 min. The activation was applied to 3 batches of carbonized carbon that did not aggregate, i.e., CMHC (15%), CMHC (8%) and CMHC (12%) samples. In real practical applications, temperatures above 800 °C are usually used and the activation process is carried out in a neutral environment with a mixture of water steam and CO_2_. Bansal & Goyal [40], Mahmodi et al. [32] and Hokkanen [35] proved that the activation temperature has a significant impact on the depth of penetration of activators into the internal structure of the carbon as well as the efficiency of activated carbon production itself, significantly reducing the time the load stays in the furnace. The activation temperature is usually between 200 °C and 1100 °C [41]. Nevertheless, some researchers report a temperature range of 400–500 °C [31,40]. However, it should always be recognized that increasing the activation temperature often results in a decrease in the yield of activated carbon batch and, at the same time, an increase in the volume of volatile substances released [12,41]. Additionally, at higher temperatures new active spots in the carbon structure are released as a result of the escape of volatile substances from the raw material. Organic material that did not react in the carbonization process is also charred. Thus, higher temperatures lead to a more stable mineralized final product [28,29,40,42]. Considering mentioned above scientists’ experience, there was taken decision to conducted tests on higher temperatures in industrial activation process.

After the process, samples were taken for testing. Some of the activated CMHC granules (15%) were incinerated, resulting in lower AC production yields. From this reason activation process for incinerated sample was repeated in 900 °C and 90 min.

Conducted tests showed (Table 5) that the AC with 15% CMHC had an BET surface 722.5 m^2^/g, Iodine Number INo of 843 mg/g, a Mechanical Strength of 98.3%, an Ash Content of 35.83% and a low Bulk Mass of 394 g/dm^3^. Unfortunately, only part of activated material may have been tested because some of it was incinerated. Due to those losses experiments were repeated with 15% CMHC at the same temperature of 900 °C but for a shorter time of 90 min. For this sample BET surface was higher and reaches 788.1 m^2^/g, the Iodine Number values obtained were lower (823 mg/g), the Mechanical Strength has improved (99.9%) and the Bulk Mass increased (428 mg/dm^3^). As there were no excessive material losses with those process parameters, the productivity of the trial increased and there were fewer granules incinerated. A CMHC content of 15% relative to 3 kg of coal feedstock appears to be quite high, which translates into a high investment cost of raw materials. However, it has been shown that with such material ratios it is possible to manufacture well-shaped granules and perform carbonization and activation processes and, as a result, it is possible to obtain activated carbon with good adsorption and mechanical properties.

It is important to remember that the primary objective of the manufacture and then activated carbons use under real conditions is to develop and fully exploit their internal pores [4,7,9]. Based on conducted research, it can be concluded that the tested coals showed a higher ash content compared to coals reported in the literature [9,28,39]. In general the process of ashing and surface preparation of AC allows full access to its surface structure. Activated carbons have pores described as micropores with diameters up to 2 nm and meso- and macropores with diameters greater than 2 nm. The presence of micropores is most desirable in an adsorbent and considerably increases its adsorption properties. According to the laws of physics [7,43], the action of electrokinetic forces inside these pores leads to the formation of active areas on their opposite walls and an increase in the adsorption capacity. Due to this phenomenon, the adsorption strength in micropores is dominant compared to the adsorption strength in mesopores. Adsorption in macropores, on the other hand, is practically negligible and the macropores themselves are simply transport pathways for contaminant particles [41,43,44,45,46,47,48]. Tested in this work ACs created in professional manufacturing conditions had quite high pore volume with the best properties for partially incinerated sample 15% CMHC. The total pores volume was only 0.58 cm^3^/g, 0.52 cm^3^/g for CMHC (12%), 0.54 cm^3^/g for CMHC (8%) and for CMHC (15%) in 90 min–0.51 cm^3^/g. It is worth to conclude that effective pore volume is not related only to temperature but also to the form of activating agent. An example may the research conducted by Sych et al. [37] on coal-based material. He showed that ACs produced by water steam-air treatment under appropriate temperature conditions lead to microporous final products with a large number of micropores with the smallest dimensions.

Inferences conducted from the activation with CMHC (15%), CMHC (8%) and CMHC (12%) allowed continue tests and activation to be carried out under industrial conditions using an professional production line. For technological tests double-stage activation of charcoal only with CMHC (8%) was used. Previously AC & CMHC (8%) was tested during single activation and showed similar parameters compared to higher concentrations of CMHC. Samples with 15% CMHC were rejected due to the high investment cost. Only the lower concentration of CMHC could be considered for real and continuous production. As before, the pretreated coal was subjected to activation with water steam and recycled carbon dioxide from carbonization process.

Grand-Activated Ltd. Company’s standards for processing hard coal require temperatures of 600–800 °C for carbonisation and 600–1000 °C for activation. The double-activation clearly improved the adsorption and strength parameters of the produced AC. The material after activation had a higher Mechanical Strength reaching 99.9% and a lower Ash content, which decreased from 18.15 to 12.84%. Together with the reduction in Ash content, the proportion of Volatile Matter decreased from 18.15 to 9.91%. This is explained by the fact that the organic material was mineralised to a greater extent as a result of the 2-stage activation. As a result, the product achieved a lower Density, which decreased by almost 200 kg/dm^3^ to 428 kg/dm^3^.

The BET surface and structural properties of the produced carbons are shown in Table 6. The activation of the tested batch of material was then repeated at a lower temperature of 600 °C and the same time of 120 min. It was also carried out in the same in-house activation furnace. As a result of activation with water steam and carbon dioxide, there was noted significant increase in the BET surface of activated carbon to 774 and 968 m^2^/g and an increase in pore volume from 0.58 to 0.82 cm^3^/g. It was also found that, as a result of the modification of the activation process, there was an increase in the diameter of internal pores for the granular carbons, and consequently a decrease in the proportion of micropores in the internal structure of the tested carbons to 50–70%. However, the obtained results indicated, that there was an overall increase in the total adsorption capacity, compared to the single-stage activation. This growth is probably due to an enlargement of the pore diameters as a result of stronger mineralization of the substrate by the two activation agents and the development of a mesoporous structure. The change in the internal surface area of the mesopores is the result of the formation of new mesopores from micropores through an increase in the diameter of the micropores during the dual activation process [8]. The contribution of micropores to the adsorption capacity decreased slightly for the studied adsorbents.

The detailed activated carbon specification is compulsory for manufacturers. There must be included precise determination of the pore distribution, with division into micropores, mesopores and macropores. In addition, the chemical structure of the carbon surface influences its adsorption properties. The nature of the surface also depends on the type, amount and mode of connection of various heteroatoms (e.g., under aqueous or thermal conditions), such as Oxygen, Hydrogen, Nitrogen, Sulphur, Chlorine and other carbon-bound atoms [49,50].

According to literature data [1,2,3,4] BET surface of the activated carbon may reach in particular controlled up to 1000–4000 m^2^/g. Different scientist conducted research on different matrix with activating agents. Jiang&Zhao [51] tested activation process which was optimized by combination of several factors considering in the process’s performance. That is why conducted experiments allowed for synergic effect joining chemical activators, temperature, activation time and extra stage of activation (double-activation). Thermal processing decrease functional groups of acidic characteristics and increase of electrokinetic potential follows this phenomena. To get even better results in carried out tests polymeric binder was added causing positive charge growth on the active surface of AC. Research on several biocarbons conducted Sipola et al. [52] and studied pore volume and surface area. He noted that BET varied from 238 to 3505 m^2^/g and chemical composition of activated carbon surface closely depended on activation process conditions. His team studied chemical activation supported by KOH and created AC with high share of micropores in comparison to mesopores and macropores. Activation with CO_2_ on the other hand helped to develop mostly mesopores and macropores. Research conducted by Zuo et al. [53] showed that carbon activated in the temperature of 400 °C had porous structure with specific surface area of over 600 m^2^/g and pores volume about 0.3 cm^3^/g. Also Kumar & Jena [54] observed surface area and pore volume for carbon activated in different temperatures. The conclusion were—higher temperature higher BET surface and higher total pores volume. In their research BET increases from 2223–2636 m^2^/g and pores volume from 1.29 to 1.53 cm^3^/g. Conducting research on activated carbon manufacturing both scientific research and industrial trials, there should be taken into account that particular activation method, activating agent and process conditions influence ACs parameters as its structure, pores volume and chemical characteristics of surface. Described in this paper results showed that manufactured AC with 15% CMHC was mainly mesoporous. Similar findings had Bubanele & Shivashankar [55] and Slasli et al. [56]. According to their experiments water steam and CO_2_ are effective and cheap activatores. Professional AC manufacture should be economically efficient to find bigger trade market. Water steam and CO_2_ let to reach internal surface area over 1000 m^2^/g [52]. Water steam react with charcoal much faster than carbon dioxide. Therefore both activated agents were joined in AC production and such combination let to reach higher BET surface and higher porosity than primary activated carbon manufactured before with molasse.

**Figure 3:**
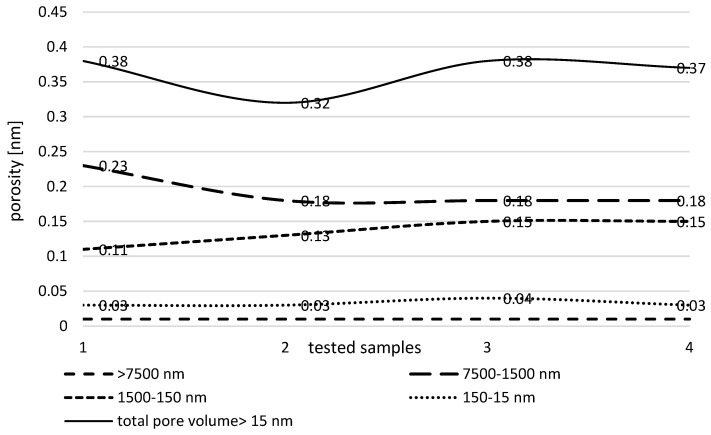
Pore distribution in carbon material after single activation. 1—sample with CMHC (15%) 120 min activation, 2—CMHC (12%) 120 min, 3—CMHC (8%) 120 min, 4—CMHC (15%) 90 min. Source: own elaboration.

**Figure 4:**
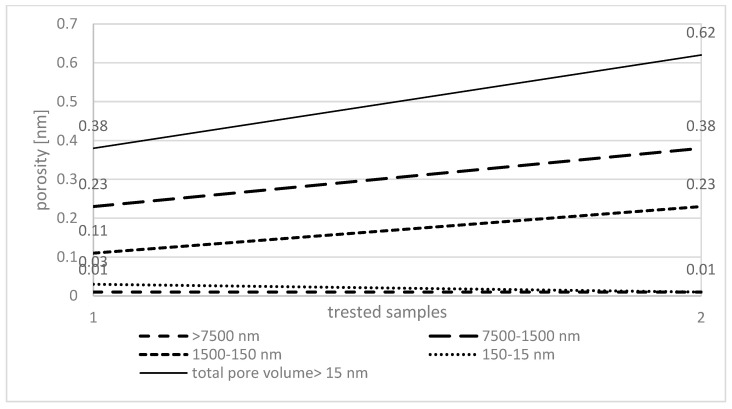
AC porosity after double-stage activation with 8% CMHC. 1—single activation for 8% CMHC, 2—double activation for 8% CMHC. Source: own elaboration.

In conducted studies there were observed changes in the pore structure of AC formed with 8% CMHC when comparing the 1-stage to the 2-stage activation. It should be noted that both activations were carried out for 120 min each, but at different temperatures of 900 °C and 600 °C. The charcoal with 8% CMHC was positively evaluated in the previous single-activation test stage, obtaining a high mechanical strength of 99.7% and a total pore volume comparable to the material with 15% CMHC. Both ACs reached similar and the best parameters. However, the higher proportion of binder was shown to affect the uncontrolled incineration of the activated product. It was therefore assumed that a 8% CMHC concentration would be the most optimal and a double activation was performed only for it. It can be confirmed by other scientific experiment conducted by Czepirski et al. [47] who carried out studies with physical AC activation (steam and CO_2_) too. He showed that the surface area of SSA, the total pore volume and the volume of the micropores and mesopores of the activated AC was significantly higher than obtained by single physical activation. On the other hand, Mendoza-Carrasco et al. [48], in his study, found that the activated charcoal using steam had an efficiency of 10% higher by double-stage than single-stage activation. His test results showed that the surface area and volume of the micropores and mesopores of the carbon tested were higher with steam activation than CO_2_ activation. Presented examples of other research may prove, the choice of activation process parameters depend mostly on the requirements of the final product. With 1-stage activation, the proportion of individual pores is shown in Figure 3, while with 2-stage activation it is shown in Figure 4.

**Figure 5:**
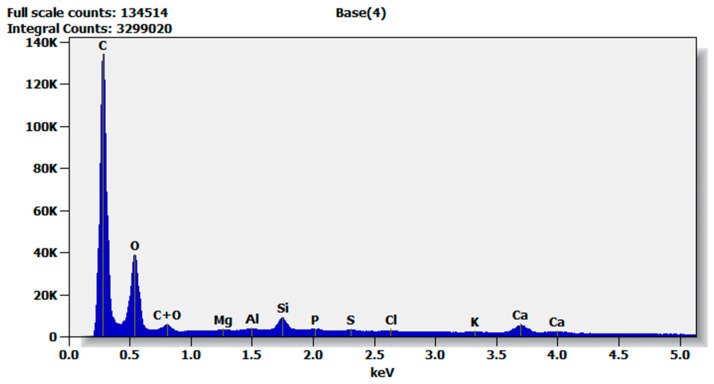
Chemical characterization of AC with 15% CMHC. Source: own elaboration.

The type of activating agent is a parameter that has a major impact on the properties of the produced AC [4]. The elemental composition of the best tested activated carbon with 15% CMHC is presented in Table 7 and Figure 5.

The part of conducted research was also analysis of outer structure of the best activated carbon selected from the group of all tested ACs. External structure was observed using SEM microscope which allows to recognize particular elements and atoms located on the studied surface. The typical feature of activated carbon is its porous internal structure and crystalline form. AC also has a characteristic chemical elemental structure of its surface. This chemical structure strongly influences the adsorption capacity of the carbon. It should be noted that the chemical structure is strongly dependent on certain heteroatoms that are chemically bonded to the surface (oxygen and hydrogen heteroatoms) [49,50].

Analysis of the adsorption properties of activated carbon also requires, in addition to the porous structure, a precise recognition of the chemical structure of the surface. The adsorption process itself is a complex process. A number of different processes take place on the surface of activated carbon. Other processes take place inside the pores of the carbon material. As a result, the physical factors considered in previous section of this work, but also the chemical factors can have a significant impact on the adsorption capacity. Both the characteristics of the chemical complexes on the carbon surface, its porous structure and, in particular, the synergy of chemical and physical conditions are important in this case [7,12,31].

Chemical analyses of the surface of our investigated AC, beside elementary carbon and oxygen, show that it contains large amounts of mineral ions, mainly calcium, silicon and aluminum. These groups can affect the surface properties in a similar way to the acidic or basic organic functional groups of the carbon surface. Among the groups with an acidic character, there are phosphate—0.22%, sulphate—0.20% and chloride ions 16%. Of the groups with a basic character, only potassium can be mentioned. The literature [11,29,33,51] reports that thermal treatment of activated carbon reduces the acidic functional groups on the surface, and this reaction is accompanied by an increase in the electrokinetic potential.

Furthermore, activated carbon produced with the addition of synthetic polymers may contain hydrophobic fragments of partially decomposed polymer on the surface. The literature [13,18,20] reports that these residues significantly affect the adsorption process of hydrocarbons in gaseous and water-soluble form. This relationship has been observed for various groups of both aliphatic and cyclic non-aromatic hydrocarbons. These impurities are retained on the carbon surface as a result of dispersion forces, which attach most strongly to the apolar part of the carbon structure.

In the group of industrial AC activators, physical factors should be mentioned first, i.e., the process temperature where physical activation is carried out by pyrolysis in an oxidising gas flow at elevated temperatures. Physical activation is the most common method of producing activated carbon, which does not require chemical additives that often significantly increase the sale price of the produced carbon [40]. Unfortunately, it requires a longer time for the material to stay in the activation furnace and is therefore more energy consuming [42]. In conducted research, at activation temperatures around 900 °C and with extended activation time or with repeated activation 2–3 times, there was increased mineralization of the carbonaceous mass observed, which was beneficial in the development of the internal surface and pore structure (Figure 4). On the other hand, according to the literature [7,28] increasing the activation temperature causes a decrease in the volume of both mesopores and micropores, in favour of the formation of macropores. Conversely, a low activation temperature does not allow the internal structure of the carbon to fully develop and results in a product with a high proportion of mesopores and a low proportion of micropores [21]. Based on presented in this paper results, the porosity of the activated material increased from 0.52 to 0.82 cm^3^/g, with the highest proportion of meso- and macropores.

Activated carbon tested in this work is intended to be directed for full production scale and implementation to usage. Carried out tests have to be repeated in technological production line and then sent to certification. Only ACs having certification of the national hygiene institute, can be distributed to the market. The best tested AC appeared to be product formed by double-stage activation technology with 8% CMHC binder. It may be useful in adsorption of organic compounds, which is essential for the treatment of water for both agricultural and drinking purposes. With this AC, metal ions can also be removed from drinking water. It will also find application in the treatment of industrial and municipal wastewater. Manufactured and described in this paper, activated carbon can be an effective adsorbent of organic substances such as oils, fats, organic chemicals or dyes, which are often present in wastewater.

## 4. Conclusions

1. Until now most granulated AC production was based on the sugar beet molasse as a binder. However, recently, due to inflation and difficult supplies other binders had to be considered. In conducted research there were chosen polymeric binders CMHC, POPE, MHPC used so far for construction or pharmaceutical industry. They have not yet been applied in AC manufacturing.
2. It was observed that the formed AC granules, using concentrations of 1% and 5% of tested polymers, were shapeless, short, fragile, deformed quickly and self-agglomerated into larger agglomerates. Only higher concentrations of polymers allowed granules to be formed. With 8, 12 and 15% CMHC, the granules were long, but stable after granulation.
3. During the carbonization tests, there was no depolymerization of the AC containing POPE- and MHPC and the granules become fluidized and then agglomerated. Only samples with 8, 12 and 15% CMHC had a high mechanical strength of approx. 99.9% and could be subjected to the activation process.
4. The first activation trials were carried out at 900 °C for 120 min. Some of the granules with 15% CMHC were incinerated, so activation of this batch was repeated at the same temperature but shorter time of 90 min. Similar parameters to 15% CMHC reaches also AC with 8% CMHC. To industrial phase of experiments there was chosen AC with 8% CMHC because of economical efficiency. Higher concentration generates a higher investment cost, which are important factor in professional manufacturing.
5. Tests with single- and double activation of AC with 8% CMHC showed significant increase in the specific surface area and pore volume: BET changed from 774 to 968 m^2^/g and pore volume form 0.63 to 0.82 cm^3^/g. Better results were reached for double-stage activation.
6. It was observed that, as a result of the modification of the activation process, an increase in the diameter of the internal pores and, consequently, a reduction in the proportion of micropores to 50–70% was observed for the studied granular carbon. However, based on the obtained results, it can be observed that there was an overall increase in the total adsorption capacity, compared to the single-stage activation. It made possible to recognize 8% CMHC as an effective AC’s binder which let to achieve high adsorption and mechanical strength parameters.
7. Chemical analyses of the investigated AC surface showed large rate of mineral ions: calcium, silicon and aluminum. Additionally groups with acidic character were recognized: phosphates—0.22%, sulphates—0.20% and chlorides ions 16%. Basic character had only potassium. Acidic groups were reduced in comparison to raw hard coal.

## Figures and Tables

**Table 1:** Properties of the used binding polymers.

	CMHC	POPE	MHPC
Molecular formula	[C_6_H_7_O_2_(OH)_2_OCH_2_COONa]_n_	(C_6_H_9_NO)_n_	C_56_H_108_O_30_
Molecular weight g/mol	250,000	35,000	6.1 × 10^5^
pH	6.5–8.5	3–7	5–8
Density g/cm^3^	750	1.2	0.410
Viscosity mPa*s	2000	5000	20,000
Melting point °C	274	180	250
Appearance	white fibrous powder	yellowish hygroscopic amorphous powder	white powder

Source: Own elaboration based on [14,24,25,26,27].

**Table 2:** Raw materials used for granulation and carbonisation at laboratory scale.

Pulp	Raw Materials for AC Processing
1.	3 kg ground coal, 800 mL water, 30 g CMHC (1%)
2.	3 kg ground coal, 800 mL water, 30 g POPE (1%)
3.	3 kg ground coal, 900 mL water, 150 g CMHC (5%)
4.	3 kg ground coal, 900 mL water, 150 g POPE (5%)
5.	3 kg ground coal, 1000 mL water, 0.2 kg molasse, 150 g CMHC (5%)
6.	3 kg ground coal, 1200 mL water, 450 g CMHC (15%)
7.	3 kg ground coal, 1200 mL water,150 g MHPC -2 (5%)
8.	3 kg ground coal, 900 mL water, 240 g CMHC (8%)
9.	3 kg ground coal, 1100 mL water, 360 g CMHC (12%)
10.	3 kg ground coal, 900 mL water, 240 g POPE (8%)
11.	3 kg ground coal, 900 mL water, 240 g MHPC-2 (8%)

Source: own elaboration.

**Table 3:** Laboratory analytical methods for activated carbon testing.

	Parameter	Standard [28]
1.	Bulk Density (BD)	PN-74/C-97554
2.	Water Absorption (WA)	PN-74/C-97554
3.	Specific Surface Area (SSA)	PN-74/C-97554
4.	Volatile Matter (VM)	PN-EN 1860-2:2006
5.	Elemental carbon (C)	PN-EN 1860-2:2006
6.	Ash (A)	PN-84/C-97555/08
7.	Moisture (M)	PN-84/C-97555/09
8.	Methylene Numbe (MNo)r	PN-82/C-97555.03
9.	Iodine Number (INo)	PN-83/C-97555.04
10.	BET surface	PN-ISO 9277
11.	Sieve analysis (SA)	PN-87/C-97555.01
12.	Pore volume * (PV)	Company own instruction manual
13.	Mechanical Strength	Company own instruction manual; PN-90/C-97554
14.	Abrasion	PN-90/C-97554; PN-EN 12915-1

* Porosimeter test. Source: Own elaboration present also in the publication [28].

**Table 4:** Basic parameters of AC in the process of carbonization.

	Batch Weight [g]	Yield [g]	Efficiency [%]	BD [g/dm^3^]	Ash [%]	VM [%]	Flash Point [°C]	Mech. Strength [%]
1	660			granules agglomerated				
2	660			granules agglomerated				
3	660			granules agglomerated				
4	660			granules agglomerated				
5	660			granules agglomerated				
6	500	327	65.4	520	7.59	3.45	318	95.9
7	660			granules agglomerated				
8	500	338	67.6	570	6.89	2.77	330	97.9
9	500	330	66	539	7.66	2.34	339	99.9
10	500			granules agglomerated				
11	500			granules agglomerated				

Source: Own elaboration.

**Table 5:** Parameters of activated carbon granules after a 1-step activation process at 900 °C and 120 min and in 900 °C and 90 min.

Parameter	120 min			90 min
	CMHC (15%)	CMHC (12%)	CMHC (8%)	CMHC (15%)
Batch weight [g]	450	450	450	450
Yield [g]	122	157	162	159
Efficiency [%]	27.1	34.8	36	35.3
Density [g/dm^3^]	394	428	430	428
Ash [%]	35.83	12.84	11.78	15.95
Water absorption [cm^3^/g]	0.37	0.44	0.41	0.57
Abrasion [%]	0.25	0.2	0.1	0.2
Granule diameter [mm]	1.54	1.88	1.74	1.84
Granule contraction rel.to dryied [%]	10.58	7.02	6.11	8.58
Volitile matter [%}	44.5	21.7	18.6	18.8
INo [mg/g]	843	853	854	823
Mech. Strenght [%]	98.3	98.5	99.9	99.9
BET surface [m^2^/g]	722.5	764.9	782.6	788.1
Pores volume [cm^3^/g]	0.58	0.52	0.54	0.53
Sieve analysis (residues) [%]				
3.5 mm	0.7	0.6	0.6	0.6
2.75 mm	19.6	18.6	16.8	17.6
2.0 mm	59.7	60.01	61.23	60.7
1.5 mm	18.4	19.7	20.05	19.2
<1.0	1.6	1.09	1.32	1.9

Source: own elaboration.

**Table 6:** Parameters of activated carbon granules after a double-stage activation process with CMHC 8%.at 900 °C and 120 min for 1st stage and 600 °C and 120 min for 2nd stage.

	1-Stage Activation	2-Stage Activation
Batch weight [g]	450	162
Yield [g]	162	135
Efficiency [%]	36	83.1
Density [g/dm^3^]	669	428
Ash [%]	18.15	12.84
Water absorption [cm^3^/g]	0.42	0.48
Abrasion [%]	0.1	0.2
Granule diameter [mm]	1.88	1.72
Granule contraction rel.to dryied [%]	8.13	7.02
Volitile matter [%}	18.15	9.91
INo [mg/g]	811	862
Mech. Strenght [%]	99.7	99.9
BET surface [m^2^/g]	774.3	968.1
Pores volume [cm^3^/g]	0.63	0.82
Sieve analysis (residues) {%}		
3.5 mm	0.6	0.3
2.75 mm	16.8	13.6
2.0 mm	61.23	68.01
1.5 mm	20.05	17.14
<1.0	1.32	0.95

Source: own elaboration.

**Table 7:** Chemical characterization of AC with 15% CMHC.

Element	Net Counts	Weight %	Atom %
C	868,222	47.30	55.57
O	262,984	48.15	42.46
Mg	3174	0.12	0.07
Al	9260	0.27	0.14
Si	61,313	1.61	0.81
P	8980	0.22	0.10
S	8413	0.20	0.09
Cl	5690	0.16	0.06
K	4327	0.14	0.05
Ca	51,794	1.85	0.65
Total		100.00	100.00

Source: own elaboration.

## Data Availability

Data are contained within the article and Supplementary Materials.

## Supplementary Materials

The following supporting information can be downloaded at: https://www.mdpi.com/article/10.3390/ma17081753/s1, the entire file containing the precisely described methodology.
