# Supplementary material for: Manufacturing Options for Activated Carbons with Selected Synthetic Polymers as Binders
Source: Materials (Basel). 2024 Apr 11;17(8):1753. doi: 10.3390/ma17081753 (PMC11051125; doi:10.3390/ma17081753)
Supplement: Supplementary file 1 [file materials-17-01753-s001.zip › materials-2926041-supplementary.pdf]

Analytical research carried out in the Research and Development Department of Grand-Activated Sp. z o. o. are implemented based on standards and internal procedures in force in the Quality Department of Grand Activated Sp. z o. o. Table 13 presents a summary of all regulations applicable in the above-mentioned company that were used for research in the article.

Table S1. Laboratory analytical methods for activated carbon testing.

	Parameter	Standard
1.	Bulk Density (BD)	PN-74/C-97554
2.	Water Absorption (WA)	
3.	Specific Surface Area (SSA)	
4.	Volatile Matter (VM)	PN-75/C-97553
5.	Elemental carbon (C)	
6.	Ash (A)	PN-84/C-97555/08
7.	Moisture (M)	PN-84/C-97555/09
8.	Methylene Number (MNo)r	PN-82/C-97555.03
9.	Iodine Number (INo)	PN-83/C-97555.04
10.	Molasses Number (MNo)	PN-86/C-97555.05
11.	Sieve analysis (SA)	PN-87/C-97555.01
12.	Pore volume * (PV)	Company own instruction manual

* Porosimeter test

Determination of bulk density of dried activated carbon

In order to determine the bulk density of activated carbon, a sample weighing approximately 150 g was taken and poured onto a sieve with a mesh diameter of 0.5 mm. It was then sifted by hand until the dust was removed. Using a spoon, coal was poured in portions into a 100 cm^3 glass cylinder. After pouring each portion of coal, the edge of the cylinder base should be tapped against the wooden table board for about 30 s, keeping the cylinder tilted at an angle of about 80º and simultaneously rotating it around its axis. After filling the cylinder to the 100 cm^3 mark, its contents were transferred to the weighing pan and weighed with an accuracy of 0.05 g. The bulk density of dry coal (X1), in grams per dm3, was calculated according to the formula:

$$X_1 = \frac{m_1\, x(100-a)}{100} x\ 10 \qquad \text{(S1)}$$

Where: m_1 – mass of 100 cm^3 of wet coal, g, a – water content in % (m/m).

Determination of water adsorption using the drying method

The activated carbon sample for water content testing was thoroughly mixed. Then, approximately 10 g of granulated or grain coal was taken from several places into a weighing vessel previously dried with a lid in a dryer to a constant mass. The coal was spread evenly on the surface of the bottom of the vessel, covered with a lid and weighed. Then the vessel was placed in an electric dryer heated to 105÷110ºC and subjected to a drying process for 2 hours. After drying, the vessel with coal was covered with a lid, removed from the dryer and left in the air for about 3 minutes, then placed in a desiccator. The sample cup was left in the desiccator until it cooled to room temperature and then weighed. Then, a 30-minute control drying was carried out and repeated until the difference in mass after two subsequent weighings was less than 0.005 g. Calculate the water content in the activated carbon test sample (X1) as a percentage according to the formula:

$$X_1 = \frac{m_1 - m_2}{m_1 - m_3} * 100 \quad \text{(S2)}$$

Where: m_1 – mass of the container with a weight of coal before drying, g, m_2 – mass of the container with a weight of coal after drying, g, m_3 – mass of the vessel, g.

Determination of Sieve analysis (SA)

The particle size distribution of melded activated carbon was performed in accordance with ISO 2591-1. Sieve analysis was performed using a single sieve or a set of control sieves depending on requirements and specifications. The analysis involves gently placing the material on a control sieve with the required mesh size or a set of sieves and separating the material by shaking and tapping into oversized and undersized particles or, in the case of a set of sieves, into dimensional fractions determined by the mesh size of the sieves used. Sieve analysis was performed manually or mechanically. The end point of the sieving process has been reached when the amount passing through the sieve or one sieve from the set in 1 min is less than 0.1% of the load mass. Determining the grain composition involved weighing 100 g of the tested coal with an accuracy of 0.1 g, then pouring the weight onto the upper sieve of the prepared set of sieves and covering it. The sample was sieved successively on each sieve until it was completely sown (the order of the sieves was from the top of the set of sieves down to the bottom). After each complete sowing, the residue from the sieve was weighed with an accuracy of 0.01 g. The mass of the residue on a particular sieve also gives its percentage content.

Determination of Mechanical Strength

The weighed for strength determination was prepared from a grain-free coal sample below the sieve appropriate for the grain size of the tested coal. The density of the bulk mass was determined to be 1/20. Then, a weight of coal was poured into a horizontal drum, 5 steel balls with a diameter of 22 mm were thrown in and the drum was closed. The drum was started for 15 minutes (the number of revolutions per minute is constant and amounts to 50 rpm). After this time, the balls were removed. The contents of the drum were poured onto a sieve (suitable for the grain size of the tested coal) and sifted by shaking manually for 1 minute. The carbon remaining on the sieve was weighed with an accuracy of 0.01 g. The mechanical strength (X_1) was calculated as a percentage of activated carbon according to the formula:

$$X_1 = \frac{m_1 * 100}{m_2} \quad \text{(S3)}$$

Where:

m_2 – mass of the sample introduced into the test dish, in g;
m_1 - mass of the sample that remained on the test sieve, in g.

Methylene number {MNo)r determination

To determine the methylene number (MNo), a methylene blue solution with a concentration of 0.12% was prepared. For this purpose, 750 cm_3 of distilled water and 2.0 g of anhydrous methylene blue C16H18N3SCl with a molecular weight of 319.85 g/mol were poured into 1000 cm^3 flasks. The solution was then mixed thoroughly and left to stand for at least 3 days. After this time, the obtained solution was filtered through a Scott G-4 funnel using a vacuum filtration set. The filtrate was poured into a volumetric flask. 1000 cm^3, topped up to the mark with distilled water and mixed, and then the density of the solution was determined with a hydrometer. Then, determine the concentration of the prepared methylene blue solution. For this purpose, a volume of 250 cm^3, pour 50 cm^3 of the tested solution, 20 cm^3 of 0.1N potassium dichromate solution and the mixture was heated to 80°C in a water bath for 5 min. After cooling, the solution was filtered into a conical flask. 500 cm^3 through a Scott G-4 funnel, then the filter was washed twice with distilled water, in 4 cm3 portions, added to the filtrate. In order to

determine MNo, a portion of activated carbon, approximately 10 g of carbon, was taken and ground on a laboratory grinder. Then, the sample was passed through a 0.071 mm sieve and dried in a laboratory dryer at a temperature of 110±5°C until constant weight was obtained. Next, 0.2 g of coal was weighed with an accuracy of 0.0002 g and transferred quantitatively to a 100 cm^3 conical flask. Then, 1 cm^3 of methylene blue solution was added from the burette, shaking the contents of the flask vigorously each time until the next portion of the solution became discolored. If LM is specified, an amount of cm^3 of methylene blue solution equal to the methylene number should be immediately added to the flask with the tested coal sample. The total shaking time on the shaker should be 5 minutes (frequency approx. 120-150). Then, the mixed sample was filtered into two test tubes on funnels with a quantitative hard filter with a diameter of 125 mm, and then the extinction of the filtrate was checked using a "Spekol" spectrophotometer manufactured by Carl Zeiss. at a wavelength of 660 using distilled water as a reference. Based on these measurements, the extinction range was established in the range of 0.07-0.085, to which the tested filtrate should correspond. The result of the determination should be the amount of cm^3 of the decolorized solution of methyl blue by the tested activated carbon, for which the extinction of the filtrate is within the assumed range of 0.07-0.085.

Determination of iodine adsorption number (INo)

The method of determining the iodine adsorption number (INo) of formed activated carbons consists in titrating the iodine not adsorbed by the carbon and calculating the iodine adsorption number (LJ) value. The apparatus and instruments used to perform the determination are: a porcelain or agate mortar or a laboratory grinder, a woven sieve with a square mesh of 0.071 mm, round-bottom flasks with a capacity of 100 ml, a mechanical shaker, soft quantitative filters with a diameter of 90 mm. The following reagents were used: hydrochloric acid (HCl) p.a., 5% solution and iodine (I2) solution p.a., c (0.5 I2) = 0.2 mol/l, sodium thiosulfate (Na2S2O3 * 5H2O) p.a., solution with c (Na2S2O3) = 0.1 mol/l and soluble starch p.a., solution 1% (m/m). To perform the LJ determination, 0.2 g of the tested activated carbon was weighed with an accuracy of 0.0002 g and quantitatively transferred to a round-bottomed flask. Then 4 ml of hydrochloric acid and 20 ml of iodine solution were added. The flask was closed with a rubber stopper, placed on a mechanical shaker and shaken for 4 min, under good mixing. After this time, the contents of the flask were filtered through a soft quantitative filter and rinsed with 50 ml of distilled water to reduce iodine residue on the filter. The entire volume of the obtained solution was titrated with sodium thiosulfate against starch as an indicator. In parallel, a blank test was performed using strictly the same amounts of reagents used
and solutions. The iodine adsorption number (LJ) was calculated in milligrams per gram of carbon according to the formula:

$$\boldsymbol{INo} = \frac{(\boldsymbol{V_3}-\boldsymbol{V_4})*\boldsymbol{C_1}*\mathbf{126{,}92}}{\mathbf{0{,}2}} \quad \text{(S4)}$$

Where:

V_3 – volume of the Na2S2O3 solution used to titrate the blank sample, ml,

V_4 – volume of sodium thiosulfate solution used to titrate the actual sample, ml,

C_1 – concentration of sodium thiosulfate, mol/l,

126.92 – mass of 1 mole (0.5 I_2) of iodine, g,

0.2 – mass of the coal sample taken for determination, g.

Ash content determination

In order to determine the ash content, the collected activated carbon sample should be dried to constant weight at 150°C. Then, in the calcined crucible, weigh such an amount of the dried sample that the expected ash content is approximately 0.1 g, the mass m_1 is recorded. The crucible was placed in a muffle furnace at a temperature of 650±25°C and left to obtain constant mass. Typically, it takes about 3 hours to ash. After the time, the crucible was placed in a desiccator and left to cool to room temperature. After cooling, the mass m_2 was weighed and recorded. The ash content X, expressed as a mass fraction of the dried material, was calculated in % according to the formula:

$$X = \frac{m_2 - m_0}{m_1 - m_0} * 100 \quad \text{(S5)}$$

Where: m_0 – mass of the empty crucible, in g, m_1 – mass of the crucible with the dried sample, in g, m_2 – mass of the crucible with the ashed sample, in g.

Determination of water absorption

In order to determine the water absorption of the formed activated carbon, the following equipment and devices were used: Buchner funnel with a diameter of 73 mm, vacuum flask capacity 500 ml, open manometer, vacuum pump, sieve with a mesh diameter of 1 mm, measuring cylinder capacity. 50 ml. The set for determining the total pore volume presented in Figure 16 consisted of: 1 - Buchner funnel with a diameter of 87 mm, 2 - vacuum flask capacity 500ml, 3 – open mercury manometer, 4 – two-corner tap, 5 – Hofmann clamp, and two tees and glass connections with a diameter of 5 – 10 mm:

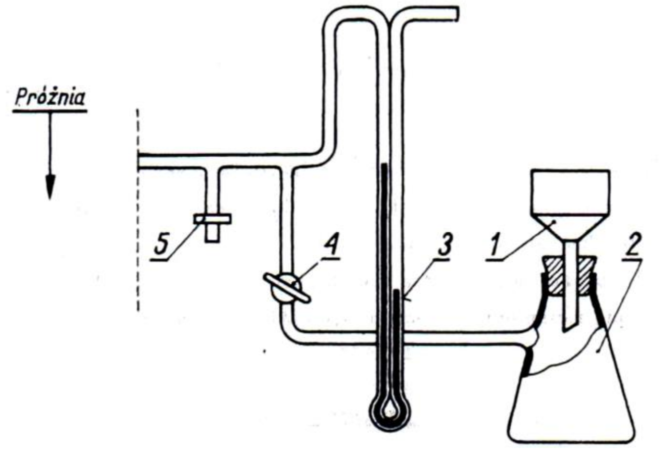

Figure S1. Kit for determining the water absorption of activated carbon
Source: Instructions for the laboratory station of Grand-Activated Sp. z o. o.

For the determination, molded activated carbon was prepared by sifting 1 mm of sample onto a sieve and then dried at 110°C for 3 hours. From the sample prepared in this way, 35 ml of coal (loosely packed) was measured using a measuring cylinder, placed in a tared vessel and weighed with an accuracy of 0.01 g. Then, the weighed coal was transferred to a conical flask and poured with 100 ml of water. The flask with its contents was heated to boiling and kept for 15 minutes. After removing the flask from the heating device, the water loss resulting from the boiling was replenished and cooled to room temperature. During cooling, a set with a vacuum pump was connected. A disk of filter paper for filtering is placed at the bottom of the Buchner funnel, closely adjusted to the diameter of the funnel. After slightly moistening it with water, it should be sucked to the bottom of the funnel by opening the tap (4) for a moment.
With the tap (4) closed, a negative pressure of 60-65 mm Hg was set on the manometer, regulated using a Hofman clamp (5). The tested coal was transferred from the flask along with water to a Buchner funnel, leveling its surface in the funnel with a porcelain spatula. The thickness of the carbon layer in the funnel should be 6 +/- 1 mm. After placing the coal in the funnel, the tap (4) was opened, simultaneously

starting the stopwatch and equalizing the vacuum pressure. The water suction time should be 5 minutes, then, without interrupting the suction, the coal was carefully poured quantitatively into a tared vessel, carefully scraping off its remnants from the filter with a spatula. In no longer than 3 minutes. After the water has been sucked out, the coal should be weighed with an accuracy of 0.01 g. Water absorption was calculated in cm^3/g according to the formula:

$$X = \frac{m_1 - m_2}{m_2 * d} \quad \text{(S6)}$$

Where:
m_1 – mass of wet coal, g,
m_2 – mass of dried coal, g,
d – water density, g/dm^3.

Determination of BET specific surface area

In order to determine the BET specific surface of the formed activated carbon, the following equipment and reagents were used: a sorption apparatus, a vacuum pump and technical liquid nitrogen. The tested sample of activated carbon was dried in a laboratory dryer at 105–110oC to a constant weight and weighed 1 g (if the area is estimated at approx. 500 m^2/g), or 0.5 g (if the area is estimated at approx. 1000 m^2 /g). Weighing was performed with an accuracy of 0.0002 g. Then, the instructions of the sorption apparatus were followed.

Examination of porous texture using mercury porosimetry

Porous texture tests are performed using the PASCAL 440 porosimeter manufactured by CE Instruments (Figure 2), in accordance with the user manual. The porosimeter operates in the pressure range from atmospheric to 400 MPa, which allows the measurement of pore volumes in the radius range from approx. 7500 nm to 1.8 nm, i.e. from macropores (7500 - 25 nm) to very small mesopores (25 - 1 .8nm)

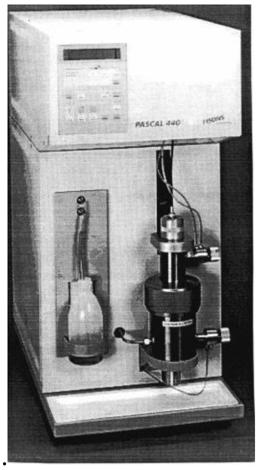

Figure S2. PASCAL 440 porosimeter
Source: Instructions for the laboratory station of Grand-Activated Sp. z o. o.

Sample degassing and mercury filling

Place the weighed sample in a measuring vessel - a dilatometer closed with a cap and a capillary tube (Figure 3). Place the dilatometer in the MACROPORES 120 degassing and filling device, turn on the pump and leave for 30 minutes.

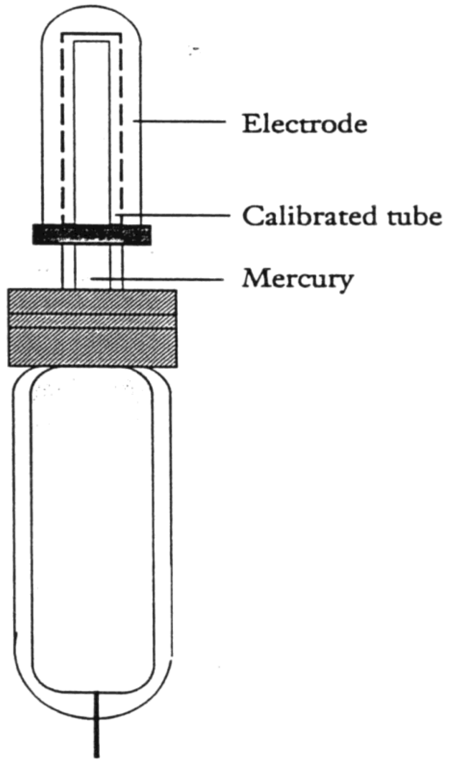

Figure S3. Mercury volume measurement system - dilatometer with capillary tube and electrode
Source: Instructions for the laboratory station of Grand-Activated Sp. z o. o.

The dilatometer is filled to the level marked on the capillary. The filling must be exactly as in the blank analysis performed to calibrate the dilatometer. The masses of the empty and mercury-filled dilatometers needed to calculate the density of the tested material are taken from a blind analysis.

Mercury penetration measurement

We weigh the mercury-filled dilatometer with the sample, fill the capillary with high-pressure oil (the one circulating in the porosimeter), and then place it in the PASCAL 440 autoclave. We start filling the system with oil and, at that time, program the analysis course. After filling the system with oil and completing programming, an analysis is performed by measuring the volume of mercury penetrating into the pores and the equilibrium pressure at which this penetration occurs.

A detailed description of the steps to prepare the PASCAL device for analysis and parameter programming is included in the analysis manual available in the laboratory.

For precise measurements, it is necessary to correct for the compressibility of oil and mercury, which is carried out by calibrating dilatometers, i.e. performing an analysis without a (blank) sample. Current blind analyzes of used dilatometers are saved in appropriate folders on the computer.

The obtained experimental data constitute the basis for calculating: specific pore volume and pore volume distribution as a function of their radii (or diameters) and other parameters characterizing the tested material: apparent density, density at maximum mercury pressure, porosity, internal pore surface. After performing the measurement, we save the analysis results in the computer's memory and, using computer software, we prepare a report containing the parameters calculated from the mercury penetration measurement. The analysis process is programmed and the experimental data are processed using MILESTON 200 software, provided by the device manufacturer. We perform the analysis twice.

Analysis results

Pore size distribution

The device records the volume of mercury penetrating into the pores of the tested material, which makes it possible to present a curve of the relationship between the volume of open pores and the applied pressure and the distribution of pore volume depending on the radii (Figure 4). Pore radii corresponding to specific pressure values are calculated using the Washburn formula (11). Due to the large dispersion of pressure values and pore radii, the results are presented in a logarithmic system. The pore size distribution can be printed in the report for any specified radius ranges.

$$\Delta\rho = \frac{4\gamma cos\theta}{d} \quad \text{(S7)}$$

Where:

d – pore diameter,

γ – surface tension of mercury,

θ – limiting contact angle of mercury,

Δp – difference of mercury pressure and gas pressure in the pores (p1 - p0).

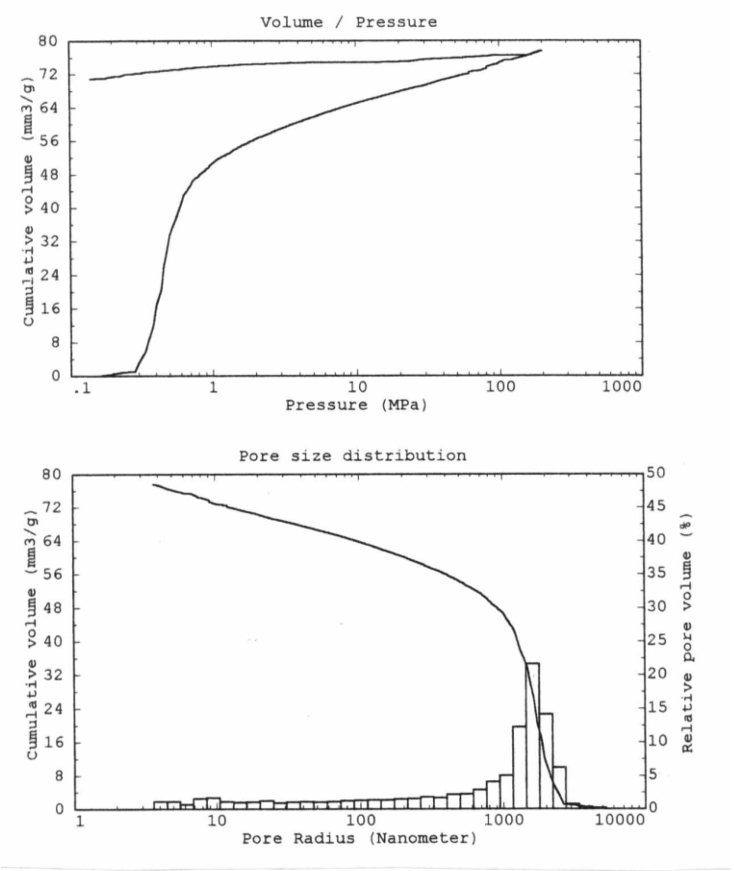

Figure S4. *Pore distribution charts*

Source: Instructions for the laboratory station of Grand-Activated Sp. z o. o.

Specific volume and density

The basic parameter determined in the analysis is the specific pore volume (on the report printout: Total cumulative volume, mm^3/g - PV), measured directly from the reduction in the mercury level in the dilatometer capillary. Mercury penetration can be used to calculate different material densities.

In the method used, the apparent density (bulk or grain density, depending on how the dilatometer is filled) is calculated based on the mass of the sample and the volume it occupies. If the dilatometer with the sample is filled with mercury under vacuum, i.e. in conditions when mercury cannot fill the intergranular spaces, the determined density corresponds to the density defined as bulk density ϱb

(Bulk density, g/cm³). Filling the dilatometer to the level marked on the capillary at atmospheric pressure fills the space between the grains, the marked density corresponds to the density of the grains (ϱp). The volume occupied by the sample in the dilatometer can be calculated as the difference in the volume of mercury in the dilatometer filled with mercury (V1) and filled with the sample and mercury (V2). By replacing the volume of mercury with the appropriate masses and density, we obtain the following relationship:

$$V_s = \frac{W_1 - (W_2 - W_s)}{\rho_{Hg}} \quad (S8)$$

and from here

$$\rho_b = \rho_{Hg} * \frac{V_s}{(W_1 - W_2 - W_s)} \quad (S9)$$

Where:
Vs – sample volume,
ϱHg – density of mercury at temperature,
Ws – sample mass,
W1 – mass of the dilatometer with mercury,
W2 – mass of the dilatometer with mercury and sample.

Apparent density at maximum pressure (Apparent density, g/cm³) is calculated from the **formula:**

$$\rho_a = \frac{1}{\frac{1}{\rho_b} - PV} \quad (S10)$$

Where:
PV - total specific pore volume, expressed in cm³/g
The calculated value differs from the actual density of the material (it is underestimated), the greater the microporosity and/or the content of closed pores of various sizes and the greater the susceptibility of the tested material to deformation under the influence of increased pressure.
Porosity
The percentage of porosity (Total porosity) is expressed as the ratio of the pore volume to the external volume occupied by the sample and calculated from the formula:

$$\mathbf{POR\% = [PV/ (1/\rho_b)] \times 100\%} \quad (S11)$$

Marking of abrasion

In order to determine the abrasiveness of activated carbon, a weigh of 100±0.01 g of molded activated carbon, free of fractions below 1 mm, was prepared and poured into the upper sieve of the set of sieves. The kit was placed on a BMD-X laboratory shaker and the kit was run for 15 minutes. After this time, the granules remaining on the sieves were weighed with an accuracy of 0.01 g. Abrasion X in % (m/m) was calculated from the formula:

$$X = \frac{(m_4 - m_5) * 100}{m_4} \quad (S12)$$

Where:

m_4 – mass of granules before abrasion, g,

m_5 – mass of granules after grinding, g,

Determination of volatile matter content

The determination of the content of volatile components was performed in accordance with the PN-EN 1860-2:2006 standard. The method involves heating a coal sample at 900°C in the absence of air for 7 minutes. The percentage of volatile matter is calculated from the mass loss of the sample minus the mass loss of moisture. To determine the content of volatile matter, the following were used: an electric muffle furnace (Figure 16), ensuring the maintenance of the set temperature (900±10°C), a cylindrical crucible with a precisely fitting lid made of fused silica. From the prepared and mixed laboratory sample, 1 g of coal was weighed with an accuracy of 0.1 mg. 1 g of coal was weighed into a crucible heated in a furnace at a temperature (900±10°C) for 7 minutes and cooled down, then the crucible was heated without a lid at a temperature of 105-110°C for 1 hour, cooled in a desiccator, the crucible with the dried sample was weighed and the crucible was closed with a lid. The crucible was placed in a muffle furnace with a set temperature of 900±10°C and heated for exactly 7 minutes. After heating, the crucible was cooled in a desiccator and weighed again. The content of volatile matter V related to the dry state, expressed as a percentage, was calculated from the formula:

$$\boldsymbol{V = \frac{(m_2 - m_3)}{(m_2 - m_1)} \cdot 100} \tag{S13}$$

Where:

m1 - mass of empty crucible, g,

m2 - mass of the crucible with the lid and the sample after drying at 105°C for 1 hour, g,

m3 - mass of the crucible with the lid and contents after heating, g.

The determination result is the arithmetic mean of the results of two parallel determinations from one laboratory sample of the tested coal.

Carbon C

The percentage of fixed carbon was determined in accordance with the PN-EN 1860-2:2006 standard. The percentage of fixed carbon was calculated from the equation: C(fix)=100-(F+A) Where: F - dry volatile matter content, %, A - dry ash content, %.
